# Urinary tract infection in the setting of vesicoureteral reflux

**DOI:** 10.12688/f1000research.8390.1

**Published:** 2016-06-30

**Authors:** Michael L. Garcia-Roig, Andrew J. Kirsch

**Affiliations:** 1 Department of Pediatric Urology, Children’s Healthcare of Atlanta and Emory University, Atlanta, GA, USA

**Keywords:** Vesicoureteral reflux, urinary tract infections, pyelonephritis, pediatric urology

## Abstract

Vesicoureteral reflux (VUR) is the most common underlying etiology responsible for febrile urinary tract infections (UTIs) or pyelonephritis in children. Along with the morbidity of pyelonephritis, long-term sequelae of recurrent renal infections include renal scarring, proteinuria, and hypertension. Treatment is directed toward the prevention of recurrent infection through use of continuous antibiotic prophylaxis during a period of observation for spontaneous resolution or by surgical correction. In children, bowel and bladder dysfunction (BBD) plays a significant role in the occurrence of UTI and the rate of VUR resolution. Effective treatment of BBD leads to higher rates of spontaneous resolution and decreased risk of UTI.

## Introduction

The prevalence of febrile urinary tract infection (UTI) in infants and children ranges from 3 to 7% and varies by age, race, sex, and circumcision status ^1– 3^. Vesicoureteral reflux (VUR) is found in 30–45% of children presenting with a febrile UTI, with an even higher risk in neonates ^4, 5^. UTI in the setting of VUR is often associated with pyelonephritis, as reflux results in direct communication of infected urine between the bladder and kidney, thus permitting cystitis to rapidly progress to acute pyelonephritis.

The diagnosis and management of VUR have been met with controversy between recent UTI guidelines published by the American Academy of Pediatrics (AAP) and the American Urological Association (AUA) reflux guidelines. Several studies have shown no significant benefit to continuous antibiotic prophylaxis (CAP) in the lowest grades of VUR, favoring a less aggressive screening and treatment algorithm. In such cases, a period of observation off CAP has been recommended. In contrast, recent large randomized controlled trials of mild to severe VUR have shown CAP and/or anti-reflux surgery to minimize recurrent pyelonephritis and, in some reports, renal scarring ^6, 7^. VUR of any grade should be viewed as one of many risk factors increasing the chance of recurrent pyelonephritis. We outline the recent advances in the medical and surgical management of UTI in the setting of VUR.

## The impact of UTI on VUR

Acquired renal scarring associated with VUR is the result of the acute inflammatory reaction that develops secondary to bacterial infection of the renal parenchyma. The inflammation is mediated by cytokine release, resulting in focal parenchymal ischemia and, ultimately, scarring ^8^. Refluxing sterile urine is not a detriment to renal function, but high-grade VUR is often associated with congenital renal dysplasia and can affect renal architecture. The extent of renal damage after a febrile infection depends on bacterial and host factors that mediate the response to infection. Late sequelae of renal scarring, such as hypertension, proteinuria, or even chronic renal failure, can be seen in the second or third decades of life ^9^.

## A shift toward observation with CAP for selected patients

VUR diagnosed in early childhood has been found to resolve spontaneously and safely in some patients after a period of observation on CAP. Prognostic calculators have been developed to predict VUR resolution in children ^10– 12^. Unfortunately, the international VUR grading system alone is subject to misinterpretation by radiologists and is over-simplistic regarding the many nuances of VUR grading. Our group recently validated a six-point scoring system based on voiding cystourethrogram (VCUG) findings that accurately predicts the likelihood of VUR resolution in children younger than 2 years old based on gender, VUR grade, VUR timing, and ureteral abnormalities ^10^. Our data, as well as others’, emphasize that low-pressure VUR (i.e. VUR occurring at low bladder volume) has a significantly lower rate of resolution when compared to the same grade of VUR occurring later in the bladder cycle (i.e. late filling or voiding VUR) ^10, 12– 14^. These recent developments provide reasonable expectations for spontaneous VUR resolution to the provider and parent based on limited imaging and patient characteristics.

The 2010 AUA’s guideline on VUR recommended goals of VUR treatment as preventing recurrent febrile UTI and renal scarring and minimizing the morbidity of treatment ^15^. A period of observation for VUR resolution is indicated given that these goals can be met. CAP has become a mainstay during this waiting period, as it reduces the rate of febrile UTI in the setting of VUR. This evidence came, in part, from the results of the Swedish reflux and the Randomized Intervention for Children with Vesicoureteral Reflux (RIVUR) trials ^7, 15^. The Swedish reflux trial randomized 203 children with grade III-IV VUR to placebo, CAP, or endoscopic injection and reported a recurrent UTI rate in girls of 19% on prophylaxis, 23% with endoscopic injection, and 57% on surveillance (p = 0.0002), while no difference was observed in boys ^6^. The RIVUR trial randomized 607 children with VUR diagnosed after febrile UTI to placebo vs. CAP and found a 50% reduction in the risk of UTI recurrence in those on prophylaxis ^7^. A subsequent meta-analysis confirmed the benefit of CAP in reducing the risk of febrile or symptomatic UTI in children with VUR (pooled odds ratio [OR] 0.63, 95% confidence interval [CI] 0.42–0.96) ^16^. A link between CAP and reduction of renal scarring is less well defined than between CAP and UTI reduction, as the RIVUR trial (inclusive of grades I-IV) and the recent meta-analysis both reported CAP use was not associated with a decrease in new renal scarring, while the Swedish reflux trial (inclusive of grades III-IV) showed a reduction in renal scarring for the CAP group ^6, 16, 17^. It should be emphasized that the genesis of renal scarring may take years to detect on renal imaging studies and that the primary outcome of the RIVUR trial was not to show a reduction in renal scarring.

## Risk factors for breakthrough UTI

**Figure 1.**
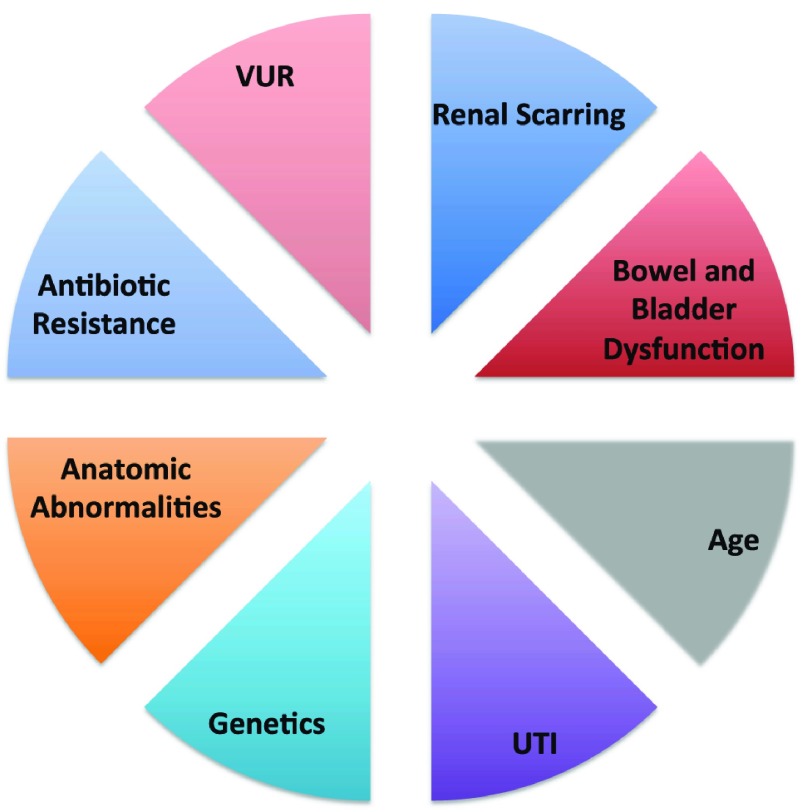
Pie graph representing risk factors for acute pyelonephritis. As shown, vesicoureteral reflux (VUR) is one of several important risk factors illustrating the multifactorial nature of urinary tract infection (UTI)/VUR management. Individual factors may or may not be present in an individual patient and play varying roles in UTI recurrence and VUR resolution and management.

Although CAP has been shown to reduce the rate of recurrent UTI, risks of recurrent pyelonephritis are not inconsequential. Factors influencing the rate of breakthrough UTI in children on CAP include a multitude of factors, as illustrated in Figure 1. Bowel and bladder dysfunction (BBD) in the setting of VUR results in a 56% risk of recurrent UTI vs. 25.4% in children with VUR only ^18^. Antibiotic prophylaxis in patients with BBD and VUR is particularly effective at reducing the risk of recurrent UTI (hazard ratio [HR] 0.21, 95% CI, 0.08–0.58) ^7^. During a period of observation, patients with dysfunctional voiding should undergo targeted BBD treatment, as this increases the likelihood of spontaneous VUR resolution (70%) compared to those with idiopathic detrusor overactivity (38%) or detrusor underutilization (40%) in patients with mild to moderate VUR ^19^.

**Figure 2.**
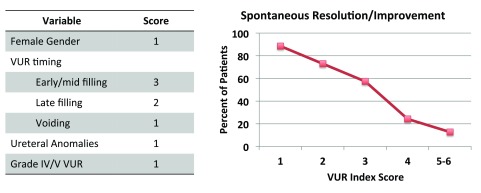
Vesicoureteral reflux (VUR) index with associated weighted scoring system predicts VUR improvement or resolution in children diagnosed with VUR <2 years old. The rate of resolution or improvement is outlined in the graph of improvement rate based on VUR index score. The graph represents an average of the initial VUR index cohort and subsequent multi-institutional VUR index validation cohort.

As stated above, the timing of VUR during a VCUG is an important prognostic marker for its resolution and risk of breakthrough febrile UTI. Alexander *et al*. identified that VUR onset at ≤35% bladder capacity on VCUG was an independent predictor of breakthrough UTI (HR 1.58, 95% CI 1.05–2.38, p = 0.03) ^13^. The likelihood of VUR resolution is also correlated with bladder volume at onset of VUR, where VUR at >50% predicted bladder capacity is more likely to resolve spontaneously (p<0.001) ^10, 11, 14^. In our recent studies, early filling VUR has been shown to be the most important predictor of non-resolution in children diagnosed before 2 years of age when calculating risk based on the VUR index ^10, 11^. The VUR index and its associated resolution rate are outlined in Figure 2. These studies taken together suggest that early filling, low-volume VUR, despite its grade, predicts both non-resolution and increased risk of acute pyelonephritis and should be the focus of further research regarding VUR management.

Parent adherence to CAP is a concern in patients with VUR, as CAP is effective only if it is given consistently. Medication adherence rates in the setting of chronic disease are relatively low, reported at 50–70% ^20– 22^. The RIVUR study documented administration of CAP at least 50% of the time in 85.2% of patients and 75% of the time in 76.7% based on parent questionnaire ^7^. However, Smyth *et al*. and Eanaretto *et al*. painted a more dismal picture regarding CAP adherence by testing urine for the presence of antibiotics with a urine positive rate of 17–67% ^23, 24^. This rate of medication adherence reflects the challenge physicians face in treating not only the disease but also the patient. The ultimate approach to VUR treatment, whether medical or surgical, involves an informed discussion with parents and that weighs all available options.

## Antibiotic choice for breakthrough UTI

Antibiotics are the mainstay of treatment for bacterial UTIs. Bacterial cystitis is more likely to predispose to pyelonephritis in the setting of VUR. Treatment is geared toward relief of symptoms through killing the offending bacterial pathogen with antibiotics. Management centers on long-established principles of antibiotic use, including confirming the presence of infection with urine analysis followed by culture and sensitivity testing and administering empiric antibiotics for common uropathogens based on local antibiograms, or previous positive urine cultures if present, and a culture-specific antibiotic once antibiotic sensitivity is available ^25^.

In the context of VUR, antibiotics are used effectively as prophylaxis against bacterial infection due to the risk of renal scarring and permanent secondary renal damage ^26^. The downstream effect of this is an increased presence of bacteria resistant to the antibiotic given in the urinary and gastrointestinal tract ^7, 27^. The temporal relationship of antibiotic exposure matters in the presence of resistant bacteria. After studying 500 children presenting with an initial UTI, Paschke and colleagues demonstrated a fourfold increased risk (OR 3.6, 95% CI 1.6–8.2) of ampicillin or amoxicillin/clavulanic acid resistance among pathogens with amoxicillin exposure in the 30 days prior to UTI; however, antibiotic exposure >60 days prior to UTI did not portend presence of ampicillin-resistant bacteria ^28^. Other authors have highlighted that antibiotic resistance occurs at a much faster rate than the decay of resistance over time ^29^. These factors argue in favor of careful consideration of targeted antibiotic choice for empiric treatment and culture-specific prophylaxis in the setting of patients with potential prolonged antibiotic exposure.

The role of prophylaxis after UTI may relate to the grade of VUR present. Several studies have concluded that prophylaxis is less beneficial in preventing UTI in children with grade I-III VUR because the baseline risk of infection is very low ^30^. However, in children with high-grade reflux, grade IV-V, the risk of UTI recurrence is increased fourfold and prophylaxis does offer significant benefit ^30, 31^. It should be emphasized that the majority of studies showing no benefit of CAP in patients with low-grade VUR included mostly grades I-II. Moderate grade III VUR has been shown to be associated with a higher risk of UTI and renal scarring compared to lower VUR grades. For example, the RIVUR trial study population consisted of 8% with grade IV VUR and no patients with grade V. Although children with grade I-II VUR were at a lower baseline risk of VUR, a reduction in recurrent UTI was noted in all groups regardless of VUR grade ^7^. Confounding the issue regarding VUR grade is the nearly 33% chance that radiologists will misgrade mild to moderate VUR and adjudication of VCUGs most often results in a higher reported grade ^7^. Furthermore, studies comparing VUR to no VUR on a single VCUG that show no difference in UTI risk may be including many patients who actually have VUR but classified otherwise, since a non-cyclic VCUG may be falsely negative in up to 20% of cases ^32^. Furthermore, the diagnosis and treatment of occult VUR (i.e. recurrent pyelonephritis despite a “normal” VCUG) has been shown to significantly reduce the incidence of acute pyelonephritis ^33^.

## The role of anti-reflux surgery in reducing breakthrough UTI

Elimination of reflux by surgical means is an effective approach to treatment. Indications for surgical correction include breakthrough UTI while on CAP, poor adherence to CAP resulting in infection, persistence of VUR after a period of observation, low likelihood of spontaneous resolution in a high-risk patient, or parent’s preference given the benefits and risks of each treatment modality. Surgical options include open or laparoscopic ureteral reimplantation or endoscopic injection of a bulking agent (dextranomer/hyaluronic acid copolymer, Deflux ^®^, is currently the only Food and Drug Administration-approved agent in the USA). Success after these procedures is defined clinically and radiographically, with clinical success being the absence of recurrent UTI after cessation of CAP and radiographically by the absence of VUR on postoperative VCUG. Radiographic success for open ureteral reimplantation is 95-98% for primary low- to moderate-grade VUR and 94% for higher-grade VUR ^34, 35^. Surgical treatment success of grade V VUR is approximately 80%. Clinical success after open surgery has been reported to be between 80 and 95% and corresponds to the rate of preoperative UTIs and presence of renal involvement on a dimercaptosuccinic acid (DMSA) renal scan or sonography ^36– 38^.

Robot-assisted ureteroneocystostomy is a relatively new procedure compared to the open approaches and fewer reports are available, with clinical success recently reported at 93% by one center ^37^ and radiographic success at 77–92% ^37, 38^. Our experience with robot-assisted extravesical reimplantation has been favorable, with greater than 90% clinical and radiographic success in a complex patient cohort including reoperative surgery for VUR and obstruction ^39^. The best approach for the patient, whether open or robot assisted, depends on patient and parent preference after a discussion of the pros and cons of each approach, such as surgical scar location and postoperative convalescence.

Endoscopic injection of dextranomer/hyaluronic acid into the subureteric space is an additional method of minimally invasive treatment for VUR. Reported clinical and radiographic success ranges from 50–93%, prompting some surgeons to avoid this method of treatment in favor of open or robot-assisted approaches ^8, 40^. The success rate is impacted by a learning curve with injection, as demonstrated by Lee *et al*., who reported an improvement from 65.9% in their first 337 ureters to 80.2% in a follow up group ^41^. Our group has demonstrated modifications in technique that resulted in a consistent 90% radiographic and 93% clinical success in children with primary grades I-IV VUR. Complex cases, such as duplex ureters or injection following failed open surgery, tend to have an approximately 10% lower success. With the double hydrodistention implantation technique (HIT), we specifically emphasize the importance of a minimum 1 ml per ureter volume of substance injected in two tandem injection points to coapt the ureteral tunnel and orifice and an objective end-point of injection being the absence of ureteral hydrodistention ^42, 43^.

After surgical correction of VUR, patients are maintained on prophylactic antibiotics until the absence of hydronephrosis on ultrasound is confirmed approximately 4–6 weeks after surgery. This protocol is followed independent of approach. Repeat VCUG is not performed at our institution after surgical correction with open or endoscopic techniques owing to the high documented success rate with open and endoscopic procedures ^44– 46^. However, postoperative VCUG is currently performed after robot-assisted ureteroneocystostomy due to the relatively new nature of this procedure. Postoperatively, patients are followed clinically for signs of infection after renal ultrasound confirms stable renal appearance and prophylactic antibiotics are stopped.

## Breakthrough UTI after anti-reflux surgery

Persistent reflux or recurrent UTI are possible after open or endoscopic surgical VUR correction, representing radiographic or clinical failure, respectively. The risk for these varies based on procedure type, as outlined above. Identification of persistent low-grade VUR after ureteroneocystostomy does not necessarily correlate with a risk of postoperative breakthrough UTI, independent of procedure type ^35, 47^. Once a patient is treated for the UTI recurrence, prophylaxis is continued until definitive treatment can be performed to avoid a recurrent breakthrough UTI. BBD should be addressed if present, as this is an independent and treatable risk factor for UTI both before and after anti-reflux surgery ^19^.

The major indication for revision surgery after corrective VUR surgery falls on the occurrence of a breakthrough febrile UTI. The surgical approach to revision surgery depends on surgeon preference. Recurrent UTI after open or robot-assisted ureteroneocystostomy can be successfully managed endoscopically or by repeat open or robot-assisted ureteroneocystostomy. Perez-Brayfield *et al*. reported the success of endoscopic injection at 88% in patients with persistent VUR after open ureteral reimplantation ^48^. Two small studies by Kitchens and Jung documented the utility of endoscopic injection following open ureteroneocystostomy, including previous cross-trigonal and extravesical approaches, with resolution occurring after one injection in 70–83% ^49, 50^.

The largest series reporting success after endoscopic re-injection is from Puri *et al*., with 1551 children (2341 ureters) undergoing primary endoscopic injection for grades II-V VUR (92.2% grade III-IV) and resolution occurring after a single injection in 87.1%, second injection in 11.3%, and third in 1.6% ^51^. The indication for repeat injection was persistent VUR, which was more common in younger children and those with a higher grade of VUR (p<0.001). Persistent VUR after repeat injection is managed by ureteroneocystostomy, which can be performed via an open approach or with robot assistance.

Robot-assisted ureteroneocystostomy in children with persistent VUR after previous anti-reflux surgery is quickly becoming a mainstay of treatment. Arlen *et al*. reported the success after robot-assisted ureteral reimplant in 11 previously treated children (10 endoscopic injection and one open reimplant) with complete resolution in all reimplanted ureters; however, one developed new onset contralateral VUR ^39^.

## Conclusion and future considerations

A shift toward observation with CAP for young patients with VUR provides an option for conservative management. Breakthrough UTI represents potential for renal damage and scarring for these patients and those who underwent surgical correction of VUR. Risk factors for breakthrough UTI and spontaneous VUR resolution have been identified, as outlined above. Future considerations for improved management of this disease include less invasive VUR diagnosis methods and non-antibiotic alternatives to UTI prophylaxis.

Current recommendations for VUR diagnosis after febrile UTI have excluded the VCUG due to its invasive nature and potential for iatrogenic UTI ^52^. The 2011 AAP UTI guidelines recommend evaluation with a renal ultrasound after the first febrile UTI and VCUG after the second. These guidelines have resulted in a substantial decrease in VCUGs and have potentially limited the use of sonograms as well ^53^. A particular concern of limiting VUR diagnosis is that many children with BBD or other urological issues will not be referred to specialists who may offer effective treatments for VUR and voiding dysfunction. While a non-invasive method for accurate VUR diagnosis would prevent patients and providers from waiting until a second episode of pyelonephritis before diagnosis ^54^, such technology is currently not readily available. Despite its many disadvantages, the VCUG remains the only test available to reliably diagnose and grade VUR.

A period of observation for spontaneous VUR resolution relies on CAP to prevent UTI recurrence and the morbidity of acute pyelonephritis and renal damage. Several arguments exist against CAP, specifically its encouragement of the development of drug-resistant bacteria and the unclear impact of antibiotics on host flora. Some reports suggest that non-antibiotic management of bacterial cystitis is effective; however, a consistent method does not exist for UTI prevention or pyelonephritis ^55, 56^. Current evidence supports traditional methods of UTI and VUR diagnosis and should continue to form the cornerstone of management until less invasive management options become available.

## Abbreviations

AAP, American Academy of Pediatrics; AUA, American Urological Association; BBD, bowel and bladder dysfunction; CAP, continuous antibiotic prophylaxis; CI, confidence interval; DMSA, dimercaptosuccinic acid; HIT, hydrodistention implantation technique; HR, hazard ratio; OR, odds ratio; RIVUR, Randomized Intervention for Children with Vesicoureteral Reflux; UTI, urinary tract infection; VCUG, voiding cystourethrogram; VUR, vesicoureteral reflux
